# The functional mechanism behind the latitudinal pattern of liana diversity: Freeze–thaw embolism reduces the ecological performance of liana species

**DOI:** 10.1002/ece3.10486

**Published:** 2023-09-19

**Authors:** Paulina Lobos‐Catalán, Mylthon Jiménez‐Castillo

**Affiliations:** ^1^ Instituto de Ciencias Ambientales y Evolutivas Universidad Austral de Chile Valdivia Chile

**Keywords:** embolism, lianas, performance, root pressure, wood anatomy

## Abstract

There is a strong decrease in liana diversity along latitudinal and altitudinal gradients at a global scale, and there is a marked difference in liana diversity between tropical and temperate ecosystems. From these observations, it has been proposed that cold temperatures would restrict the ecological patterns of liana because of their vascular system's vulnerability to freeze–thaw embolism. Our objective was to establish the functional mechanism that drives the loss of liana diversity along a latitudinal temperature gradient. We evaluate the ecological performance of liana in 10 different species based on the apical growth rate, as well as functional traits associated with efficiency (maximum hydraulic conductivity and percentage conductivity lost) and safety of water transport (vessel diameter, vessel density, wood density, and root pressure). We found that at the colder (more southern) site within the latitudinal gradient, liana species showed lower performance, with a fivefold decrease in their apical growth rate as compared to the warmer (more northern) sites. We postulate that this lower performance results from a much lower water transport efficiency (26.1‐fold decrease as compared to liana species that inhabit warmer sites) that results from higher freeze–thaw (37.5% of PLC) and reduction of vessel diameter (3 times narrower). These results are unmistakable evidence that cold temperature restricts liana performance: in a cold environment, liana species exhibit a strong decrease in performance, low efficiency, and higher safety of water transport. Conversely, at warmer sites, we found that liana species exhibit functional strategies associated with higher performance, higher efficiency, and lower safety of water transport capacity. This trade‐off between efficiency and safety of water transport and their effects on performance could explain the latitudinal pattern of liana diversity.

## Graphical abstract

Establish the functional mechanism behind the loss of liana diversity along a latitudinal temperature gradient.

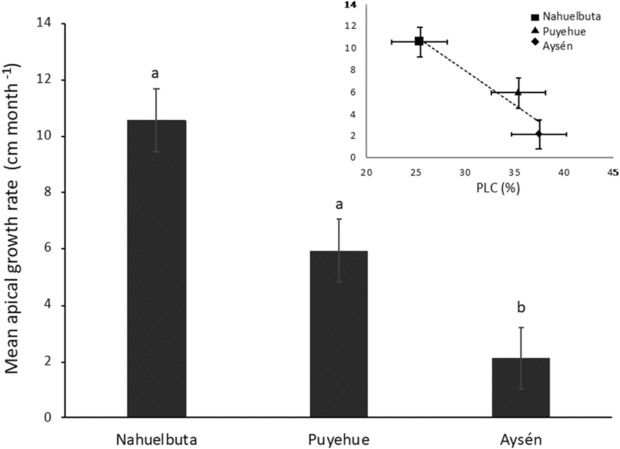

## 1 INTRODUCTION

Liana are woody climbing plants (Gentry, 1991) that contribute to the diversity, structure, and productivity of forests worldwide (DeWalt et al., 2010, 2015; Hegarty & Caballe, 1991; Putz & Mooney, 1991; Schnitzer, 2015; Schnitzer & Bongers, 2002, 2011). However, their diversity strongly decreases from tropical to temperate ecosystems (DeWalt et al., 2010, 2015; Gentry, 1991; Phillips & Miller, 2002; Schnitzer & Bongers, 2002; Schnitzler et al., 2016). The global pattern of liana diversity decreases sharply toward higher latitudes (DeWalt et al., 2015; Gentry, 1991; Hu et al., 2010; Lobos‐Catalán & Jiménez‐Castillo, 2019; Phillips & Miller, 2002; Schnitzer & Bongers, 2002; Schnitzler et al., 2016). In tropical forests, lianas represent about 25% of woody plant species (Chave et al., 2009; Gentry, 1991), while in temperate forests they make up only 10% of woody flora (Gentry, 1991), representing a fivefold decrease from tropical to temperate forests (Gentry, 1991; Schnitzer, 2018; Schnitzer & Bongers, 2002).

From this pattern, which we call the “Global Pattern of Liana Diversity” (hereafter GPLD), it has been proposed that lianas' climbing habit is incompatible with cold environments (Ewers et al., 1997; Gentry, 1991; Hu et al., 2010; Londré & Schnitzer, 2006; Schnitzer & Bongers, 2002), where they would have low survival and/or performance (Ewers et al., 1991; Jiménez‐Castillo & Lusk, 2013; Schnitzer, 2005; Sperry et al., 1987) and therefore there would be low diversity of liana species. This so‐called “cold hypothesis” has been supported by evidence of negative relationships between liana diversity and cold gradients, the observed decrease in liana diversity along altitudinal gradients (Bhattarai & Vetaas, 2003; Schnitzler et al., 2016; Vázquez & Givnish, 1998), and the parallels between latitudinal and altitudinal distribution patterns of lianas species in temperate forests (Jiménez‐Castillo et al., 2007).

The proposed underlying mechanism of GPLD is that lianas' vascular system would lose its functionality in cold environments. On the one hand, lianas have significantly higher growth rate than coexistent trees in tropical ecosystems (Schnitzer, 2005), supported by a very efficient vascular system formed by wide vessels (Davis et al., 1999; De Guzman et al., 2016; Ewers & Fisher, 1991; Gartner et al., 1990; Holbrook & Putz, 1996; Jiménez‐Castillo & Lusk, 2013). On the other hand, in cold environments, wide vessels are highly prone to freeze–thaw embolism (Ewers et al., 1991; Sperry et al., 1987; Sperry & Sullivan, 1992; Tibbetts & Ewers, 2000), which would negatively affect the vascular system's performance. Thus, the same traits that convey high efficiency of water transport in tropical forests would become maladaptive in temperate forests (Ewers, 1985; Ewers et al., 1991; Jiménez‐Castillo & Lusk, 2013; Sperry et al., 1987). The hypothesized mechanism behind GPLD is therefore based on these two lines of research: first, that the latitudinal and altitudinal gradients of liana diversity are negative related to cold gradients (DeWalt et al., 2010; Gentry, 1991; Jiménez‐Castillo et al., 2007; Lobos‐Catalán & Jiménez‐Castillo, 2019; Phillips & Miller, 2002; van der Heijden & Phillips, 2008; Vázquez & Givnish, 1998); second, that the vascular system of lianas becomes dysfunctional in cold environments (Davis et al., 1999; Ewers et al., 1991, 1997; Jiménez‐Castillo & Lusk, 2013; Sperry et al., 1987). Although these inferences have supported the hypothesis about temperature as the environmental factor limiting lianas' ecological patterns, it is imperative to evaluate the proposed mechanism in natural ecosystems.

If cold strongly limits the ecological performance of lianas toward higher latitudes, how can they even inhabit temperate ecosystems? In cold environments, lianas would confront a trade‐off between efficiency and safety of water transport capacity. Since wide vessels are highly prone to freeze–thaw embolism (Ewers et al., 1997; Zimmermann, 1983), the selection of narrow vessels would confer safety in cold environments (Carlquist, 1988; Ewers & Fisher, 1991). Another safety strategy is to reverse embolism by generating high root pressure (Sperry et al., 1987; Tibbetts & Ewers, 2000), which would be triggered by low temperatures. It has been observed that only 3 of 29 liana species generate root pressure in tropical forest (Ewers et al., 1997), while all liana species have root pressure twice as high as that of coexisting tree species in southern temperate forests (Jiménez‐Castillo & Lusk, 2013). However, any of these mechanisms would reduce the performance of liana in cold environments because narrow vessels decrease the efficiency of water transport, and root pressure implies costs because it is osmotically generated (Isnard & Silk, 2009; Jiménez‐Castillo & Lusk, 2013; Tibbetts & Ewers, 2000). These traits of avoidance and/or reversal of the embolism would not be mutually exclusive; rather, both would allow liana species to persist under low‐temperature conditions.

To study the underlying mechanism of ecological patterns, ecologists have increasingly studied the functional traits to understand species' performance from a mechanistic approach and infer niche‐based mechanisms to explain community patterns (Cadotte et al., 2015). Thus, studying a set of quantifiable traits and assessing at the level of the individual (morphological, physiological, phenological) whether they would have a direct effect (performance traits) or an indirect effect (functional traits) on the individual's biological fitness (Violle et al., 2007) would allow for the construction of general models that facilitate the understanding of the ecological strategies of the species (Lavorel & Garnier, 2002; Poorter et al., 2010; Reich et al., 2003; Westoby & Wright, 2006).

In this context, we aim to establish the functional mechanism related to liana species performance along a latitudinal temperature gradient. We addressed the following research questions: (i) Does the effect of mean minimum temperature (cold) on apical growth rate vary by site along the latitudinal temperature gradient? At warmer (more northern) sites, we predict a higher apical growth rate in liana species, whereas at colder (more southern) sites, we predict a lower apical growth rate in the liana species. Along the latitudinal temperature gradient (ii) does the effect of mean minimum temperature on functional traits vary by site? At warmer (more northern) sites, we predict a higher efficiency and lower safety in water transport traits, such as wide vessel selection, high hydraulic conductivity, low wood density, low root pressure, and low PLC. At colder (southern) sites we predict higher safety of water transport traits such as narrow vessel selection, high wood density, low hydraulic conductivity, high root pressure, and high PLC. We predict that these traits would explain the variation in the performance of liana species along this latitudinal temperature gradient. (iii) Is there evidence of a trade‐off between safety and efficiency of water transport capacity defined by functional traits in liana species, and does growth rate vary along these axes of trait variation? We predict that growth rate will be positively associated with efficiency (wide vessel selection, low wood density, high hydraulic conductivity, low root pressure, and low PLC) but negatively associated with the safety of water transport (narrow vessel selection, high wood density, low hydraulic conductivity, high root pressure, and high PLC), and these patterns will be opposite between the warmer (north) and colder (southern) sites. (iv) Is there evidence that the distribution of xylem vessel diameter is correlated with hydraulic conductivity? In order to favor the security of water transport, we expect that as the liana species inhabit colder sites, there would be a selection of smaller xylem vessels, leading to less hydraulic conductivity but generating xylem vessels less vulnerable to freeze–thaw embolism.

## 2 METHODS

### 2.1 Study area

We conducted our research in the temperate rainforests of South America, which spans a narrow coastal strip between the Pacific Ocean to the west and the southern Andes Mountains to the east, from 37° to 48° South. The climate is maritime temperate, with frequent sub‐zero temperatures during winter and spring (Di Castri & Hajek, 1976). The vegetation is mature Valdivian rainforest (Veblen et al., 1983) dominated by the tree species *Aextoxicon punctatum* R. et P. (Aextoxicaceae), *Eucryphia cordifolia* Cav. (Cunoniaceae), *Nothofagus dombeyi* Mirb. Oerst (Fagaceae), *Gevuina avellana* Mol. (Proteaceae) and *Luma apiculata* (DC) Burret (Myrtaceae). The forest canopy averages about 30 m in height, with emergent *N. dombeyi* attaining c. 40 m. All liana species frequently reach the forest canopy, and they all are evergreen. The study sites contain most of the lianas that have been described in the region, including *Berberidopsis corallina* Hook. f. (Berberidopsidaceae), *Boquila trifoliolata* (Dc.) Dcne (Lardizabalaceae), *Cissus striata* R. et. P. (Vitaceae), *Hydrangea serratifolia* (H. et A.) F. Phil. (Hydrangeaceae), *Elytropus chilensis* (A.DC.) Muell. Arg. (Apocynaceae), *Mitraria coccinea* Cav (Lardizabalaceae), *Campsidium valdivianum* (Phil.) Skottsb (Bignoniaceae), *Muehlenbeckia hastulata* je sm (Poligonaceae), *Griselinia ruscifolia* clos Ball (Griseliniaceae) and *Lardizabala biternata* Ruiz & pav. (Lardizabalaceae). In general, the temperate rainforest soil is derived from volcanic ash (andisols) (Luzio & Alcayaga Casali, 1992) and is characterized by low density, high clay content, high soil moisture, high organic matter content, and high phosphorus retention (Godoy et al., 2014). We selected three study sites along a latitudinal gradient between 37° S and 45° S. The northern site was “Nahuelbuta” (37°42′ S, 73°13′ O; 176 m a.s.l.) where the mean annual temperature is 16°C and mean minimum temperature is 4.8°C, is characterized by loamy soils (Luzio & Alcayaga Casali, 1992) with low fertility and low water content. The intermediate site was “Puyehue” (40°39′ S, 72°10′ O; 301 m a.s.l) where the mean annual temperature is 10°C and mean minimum temperature is 3.3°C, and the soil is highly porous with high organic matter content (Godoy et al., 2014), classified as andisoil. The southern site was “Aysén” (45°27′ S, 72°44′ O; 10 m a.s.l) where the mean annual temperature is 8°C and mean minimum temperature is 2.7°C (Table 1), and the soil is characterized as histosol (Luzio & Alcayaga Casali, 1992).

**TABLE 1:** Climatic variables of the three study sites. They include: average annual precipitation (mm), seasonality (number of months in which the precipitation is less than 100 mm), mean maximum temperature (°C), mean minimum temperature (°C), absolute minimum temperature (°C), number of frost events (number of days in which temperatures lower than 0°C) and frost hours (number of hours in which the recorded temperature is less than 0°C). These data were collected for 2013 (year in which we conducted the measurements) from the database of the General Directorate of Water (DGA) of Chile.

Site	Annual precipitation (mm)	Seasonality (no. month)	Temperature (°C)			No. frost events	Hour of frost
			Mean maximum	Mean minimum	Absolute minimum		
Nahuelbuta	1290.1	7	16.2	4.8	−3.2	56	237
Puyehue	3521.8	6	14.3	3.3	−5.8	81	260
Aysén	1968.8	2	13.4	2.7	−7	54	359

We assessed the performance of liana species at each site by measuring the apical growth rate (AGR) during the growing season. To establish the trade‐off between efficiency and safety of water transport, during winter, we measured maximum specific hydraulic conductivity (*K*_s_max_), percentage of loss conductivity (PLC), root pressure (*P*_x_), vessel diameter (*V*_D_), vessel density (*V*_DEN_) and wood density (WD). We measured these traits in seven species in Nahuelbuta, five species in Puyehue, and four species in Aysén, which corresponded to the maximum number of liana species found on each study site (Table S2), with 10 species studied in total.

### 2.2 Performance

To estimate the apical growth rate (AGR), we installed a mark at 5 cm below the stem apical meristem, which remained on the plant for 6 months (beginning of spring to autumn). After this time, we measured the length of the stem from the mark to the tip of the apex, subtracting the initial 5 cm. Thus, we measured the total growth of the period and averaged it over the time period (cm/month). We measured 25–30 individuals per species at each study site (*n* = 310), selecting individuals that were separated by at least 10 m, with just one apex, an average height of 60–80 cm, a 5–10 cm stem diameter, and which climbed different trees and had no visible connections.

### 2.3 Hydraulic conductivity and freeze–thaw embolism

We estimated the efficiency of water transport during winter using the specific hydraulic conductivity (*K*_s_max_) and the percentage of loss conductivity (PLC) as a proxy for freeze–thaw embolism. We collected stems from 10 individuals per species (*n* = 160), with an approximate stem diameter of 5–10 cm and an approximate height of 3 m. These were different individuals from those whose growth rate was measured, but individuals with similar phenotypic characteristics. All samples were collected during winter, in a sampling period range of 5–7 consecutive days, and the individuals before dawn to avoid xylem tension. To avoid the “open vessels effect” (Choat et al., 2010), we transported the samples in water to the location where measurements were subsequently taken. We cut the samples underwater to a length equivalent to the maximum vessel length of the species.

The specific hydraulic conductivity corresponds to the water flux rate mobilized by a driving force through a branch, normalized by the length of the segment (Tyree & Ewers, 1996), calculated as:

(1) $$\begin{array}{c} K_{S}=\mathrm{FL}/\left(\Delta {\mathrm{PA}}_{x}\right)\\ K_{S}=\mathrm{kg}\;s^{-1}m^{-1}{\mathrm{MPa}}^{-1} \end{array}$$

where *F* is the water flow mobilized through the segment expressed in mass (kg s^−1^), *L* is the length of the segment (m), Δ*P* is the pressure difference between the ends of the segment (MPa^−1^), and *A*_x_ is the xylem transversal area (m^−2^), which makes the measurements of water transport comparable between segments of different sizes. *K*_s_ was measured using a field system based on a pressure drop flowmeter (see detailed description in Brodribb & Feild, 2000; Tyree et al., 1993, 1994).

Once we determined the specific hydraulic conductivity in natural winter conditions (*K*_s_field_), we flushed out the potential embolism in the sample by refilling the segment with KCl solution for 10–15 min (Sperry et al., 1987) and carried out a new measurement of *K*_s_max_, which represents the maximum water transport capacity of the branch segment. Then, we calculated PLC as:

(2) $$\mathrm{PLC}=\left[\left(K_{s\_\max}–K_{s\_\text{field}}\right)/K_{s\_\max}\right].$$

### 2.4 Vascular anatomy

After we conducted all field measurements, we labeled a piece of each stem segment, stored it in a plastic bag, and took it to the laboratory for vessel diameter and density measuring (*n* = 160). We cut xylem transverse sections freehand with a surgical blade, observed them under a light microscope (Moticam 2500, Motic), and photographed them with a digital camera. We recorded vessel density and diameters in a continuous transect from the pith and bark of the sample. We calculated the diameter of each vessel by averaging the longest and shortest axes across the lumen (Ewers et al., 1991) using ImageJ (National Institutes of Health, USA). We then calculated an average for each branch (*n* > 100 vessels) and an average for each species (five branches). We calculated vessel density as the number of vessels per unit of area tissue in the stem transverse section. We grouped vessels in 10‐μm‐diameter classes following the methodology described by Gorsuch et al. (2001) and calculated the relative contribution of each class to total hydraulic conductivity using Hagen–Poiseuille's law, as the sum of the fourth powers of all vessel radii in the class divided by the sum of the fourth powers of all vessel radii.

### 2.5 Wood density

We determined the wood density for each stem used in hydraulic and vascular anatomy measurements (*n* = 160) by calculating the volume of the stem sample (~2 cm in length per sample) using the method of water displacement (Osazuwa‐Peters & Zanne, 2010). Then, we dried the sample in an oven for 48 h at 60°C. Thus, we determined wood density (g cm^−3^) as the dry mass divided by the volume of wood.

### 2.6 Root pressure

We estimated root pressure with bubble manometers on five individuals of each liana species (*n* = 80) as described by Ewers et al. (1997). The night prior to measurement, we cut one lateral branch and attached a manometer with a tight‐fitting vinyl tube. Each manometer consists of a glass tube of 1 mm internal diameter, closed at the distal end, filled with distilled water in its basal half, and an air bubble in its distal part. We measured the length of the bubble at pre‐dawn (*L*_pd_, in m) and then immediately cut the vinyl tubing and measured the bubble length again, thus measuring the atmospheric pressure (*L*_atm_, in m). We estimated root pressure (*P*_R_, in kPa) using the ideal gas law (Ewers et al., 1997; Tibbetts & Ewers, 2000) as:

(3) $$P_{R}=100\;\left[\left(L_{\mathrm{atm}}/L_{\mathrm{pd}}\right)-1\right].$$

We estimated the maximum height for reverting embolism (*h*_c_) by a given root pressure near the stem base (PR_b_, in kPa) (Ewers et al., 1997; Tibbetts & Ewers, 2000) as follows:

(4) $$h_{c}=\left(P_{r}-P_{c}\right)/10.$$

### 2.7 Statistical analysis

#### 2.7.1 Effect of phylogeny on functional traits variation

Before implementing models to evaluate our four key questions, we first assessed the effect of species' phylogeny on the variation in functional traits, fit a series of simple linear regressions for each study trait versus site and growth rate, with and without a phylogeny of the liana species in our data set. We evaluated the effect of phylogeny on variation using Bayesian Mixed Linear Models implemented in the package MCMCglmm v1.10 (Hadfield & Nakagawa, 2010) in R Core Team (2020). This analysis allows for the inclusion of phylogeny as a covariance matrix of random effects in a mixed linear generalized model (GLMM). We obtained this covariance matrix based on the phylogenetic tree of the focal species which we constructed from the phylogenetic distance between their genera. We compared models that include the phylogenetic covariance matrix as a random factor with a model without the phylogenetic covariance matrix. In all MCMCglmm analyses, we used 110,000 iterations, with 10,000 burning, and 100 intervals with non‐informative priors. In the prior, the residual variance was set to 1 with v = 0.002, as recommended in Hadfield (2014) for a Gaussian distribution. We compared models with and without phylogeny using the Deviance Information Criterion (DIC) (Spiegelhalter et al., 2002). We used the recommended limit of ΔDIC >10 to reject the null hypothesis (Hadfield & Nakagawa, 2010). For all models, we did not detect an effect of phylogeny (all ΔDIC <10; Table S3). All subsequent analyses were conducted using parametric linear models.

#### 2.7.2 Performance and functional trait variation in relation to cold temperature

To assess the response of liana species' performance along the temperature gradient (Question 1) we asked whether the effect of temperature on growth rate varied by site. We did this using a model of apical growth rate (performance) versus study site (location) and mean minimum temperature via ANCOVA. We next evaluated the second research question—whether the effect of temperature on functional traits (hydraulic transport, wood anatomy, and root pressure) varied by study sites—using a model of trait values versus study site (location) and temperature via ANCOVA. We fit these models using the lm () function in R, followed by the Anova () function in the car package in R Core Team (2020). We also conducted a Tukey post hoc analysis to identify the difference between sites, using the function glht in the package multcomp in R Core Team (2020). Our models met all assumptions including the independence between explanatory variables, the homogeneity of variance (Levene test in car library), and a linear relation between the explanatory and response variables. To evaluate our third research question about how the efficiency and safety of water transport relate to performance, we implemented a two‐step process. First, we identified two major axes of variation among our functional traits that correspond to efficiency and safety using principal components analysis on scaled and standardized data, via the prcomp () function in R. Having identified the two major axes, we then implemented ANCOVA analyses, asking if the effect of the principal component axis varied by site. We used lm () and ANOVA () as above. Finally, in order to answer our fourth question, we compared the frequency distribution of the xylem vessel diameters (μm) as a proxy for cold tolerance, to the distribution of total hydraulic conductivity (%) via a Kolmogorov–Smirnov test, using the ks. test function in R Core Team (2020).

## 3 RESULTS

**FIGURE 1:**
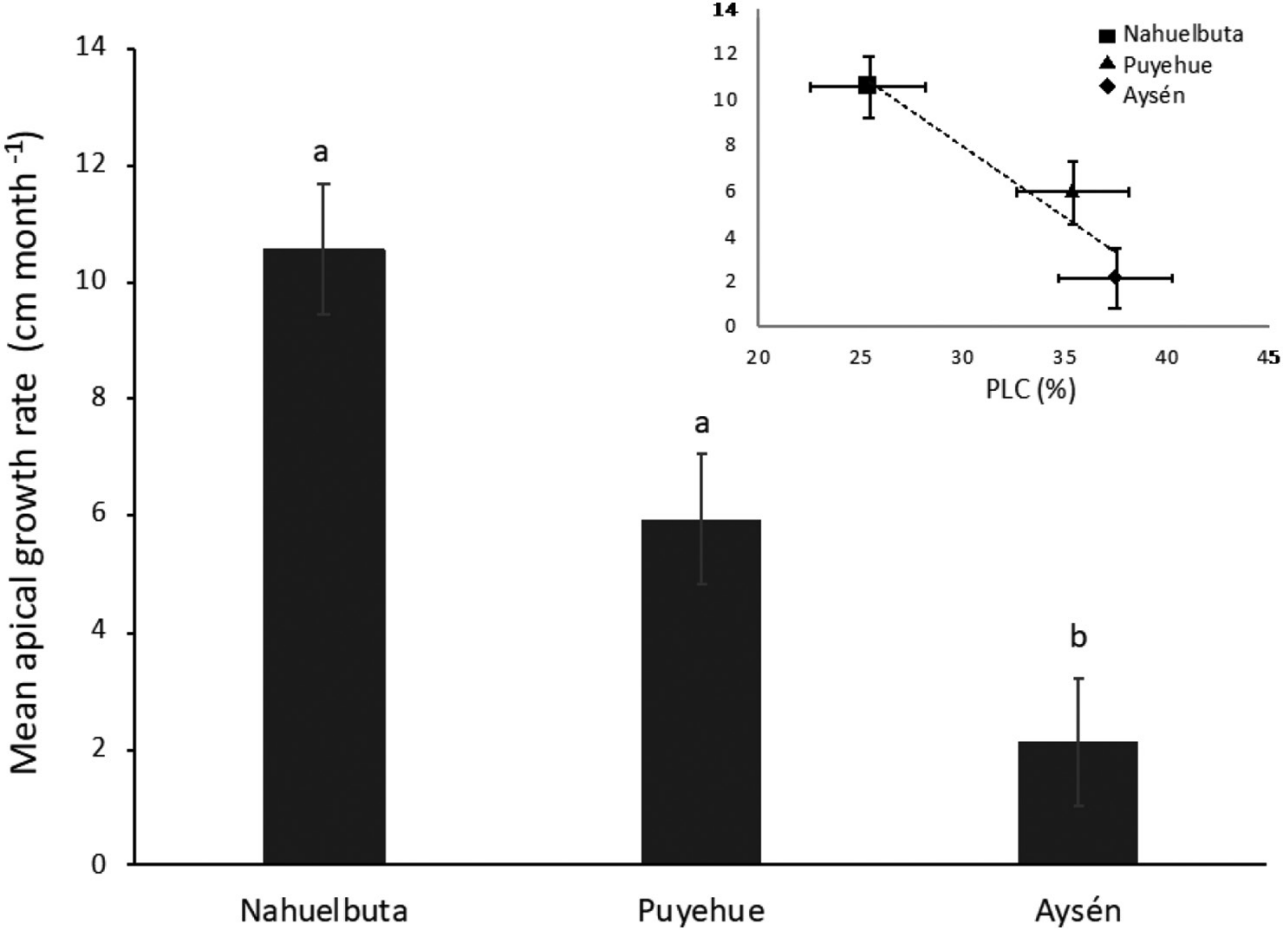
Mean apical growth rate for each study site was significantly different (*F* (2,13) 2.37 *p* = .015); “Aysén” was significantly different from “Nahuelbuta” and “Puyehue” (Tukey's HSD *p* = .017). Internal plot: Correlation between apical growth rate (cm month^−1^) and the percentage of loss conductivity (PLC %) mean per site (*R* ^2^ = .91 y *p* = .001). Bars show standard error and different letters show significant differences between sites.

There was a significant reduction in liana performance toward higher latitudes (*F* (2,13) 2.37 *p* = .015, Figure 1), reaching differences up to five times on apical growth rate between liana species at the northern and southern sites (Nahuelbuta 10.6 cm*month^−1^ v/s Aysén 2.1 cm*month^−1^, Tukey HSD *p =* .017). At the same time, the loss of liana performance was correlated with the increase in the percentage of conductivity lost toward higher latitudes (Figure 1), evidencing that cold temperatures have a negative effect on liana performance at higher latitudes.

We observed marked differences in hydraulic traits associated with efficiency in water transport capacity along the latitudinal gradient. The maximum specific hydraulic conductivity (*K*_s_max_) significantly differs between sites (*p* = .006), decreasing 26.1 times in liana species at the northern site “Nahuelbuta” compared to the southern site (“Aysen”) (Table 2, Tukey HSD *p* = .001) and the middle site “Puyehue” (Tukey HSD, *p* = .027). Nahuelbuta liana species have a *K*_s_max_ of 28.7 kg s^−1^ m^−1^ MPa^−1^ while in Aysén they barely reached 1.1 kg s^−1^ m^−1^ MPa^−1^ (Table 2). It must be added that the percentage of loss conductivity (PLC) along the sites differs significantly (*p* = .01), increasing from 24.5% at the northern site to 37.5% at the southern site (Table 2, Tukey HSD, Nahuelbuta differ from Puyehue *p* = .05 and Aysen *p* = .04). The loss of hydraulic efficiency intensified in the colder extreme of the gradient (Table 2); thus, the joint reduction in hydraulic transport capacity between the liana species in Nahuelbuta and Aysén was 42 times.

**TABLE 2:** Mean and standard error of hydraulic traits, wood anatomy, wood density, and growth rate of liana species at our three study sites.

Trait	Unit	Nahuelbuta	Puyehue	Aysén	Response	*p*
Growth rate	cm month^−1^	10.6 ± 1.2	5.9 ± 1.5	2.1 ± 2.09	LR	.015
Percentage of loss conductivity	%	25.4 ± 4.7	35.5 ± 5.6	37.5 ± 5.2	SR	.01
Specific hydraulic conductivity	kg s^−1^ m^−1^ MPa^−1^	28.7 ± 2.9	15.09 ± 3.5	1.1 ± 3.9	SR	.006
Vessel diameter	μm	68.8 ± 3.5	55.6 ± 3.3	21.9 ± 3.6	LR	.04
Vessel density	No. vessel/area	148.04 ± 88.1	264.2 ± 136.7	1090.9 ± 174.8	LR	.044
Wood density	g cm^−3^	0.38 ± 0.02	0.37 ± 0.026	0.47 ± 0.02	LR	.04
Root pressure	kPa	9.9 ± 4.8	32.7 ± 4.6	13.6 ± 5.3	SR	.014
Critical height PLC reversal	m	0.9 ± 0.35	3.2 ± 0.88	1.3 ± 0.13	SR	.03

*Note*: We highlighted the response of the traits to environmental variables, where SR (short‐term response) and LR (long‐term response) sensu Chave et al. (2009). The significance corresponds to the ANCOVA test of the difference of the traits between sites and considering mean minimum temperature as a covariable (complete test is shown in the section 3).

We also detected differences in traits associated with hydraulic transport safety. The lower water transport capacity of lianas at higher latitudes was also correlated with the decrease in xylem vessel diameter, which decreased by 30% toward the southern site (Table 2). This vessel density also differs between sites (*p* = .04), increasing by 80%, and wood density differs between sites (*p* = .04), increasing 19% toward the south end of the gradient (Table 2). As previously mentioned, water transport capacity is strongly modulated by freeze–thaw embolism, which would have a strong impact on the relative growth rate.

Contrary to what we expected, root pressure did not increase with latitude (Table 2, Tukey HSD “Nahuelbuta” only differs from “Puyehue” *p* = .02). Liana species in Puyehue showed the highest root pressure of any site along the gradient (32.7 kPa), followed Aysén (13.7 kPa) and Nahuelbuta (9.9 kPa). Based on this, lianas would be able to revert embolism up to a maximum height of 1 m in Nahuelbuta, 3.2 meters for species in Puyehue, and 1.4 meter for species in Aysén, so root pressure would have a significant effect only on the basal part of the individual. However, we might have underestimated the root pressure for the species in Aysén, since the measurements had to be repeated due to manometers freeze on a few occasions.

**FIGURE 2:**
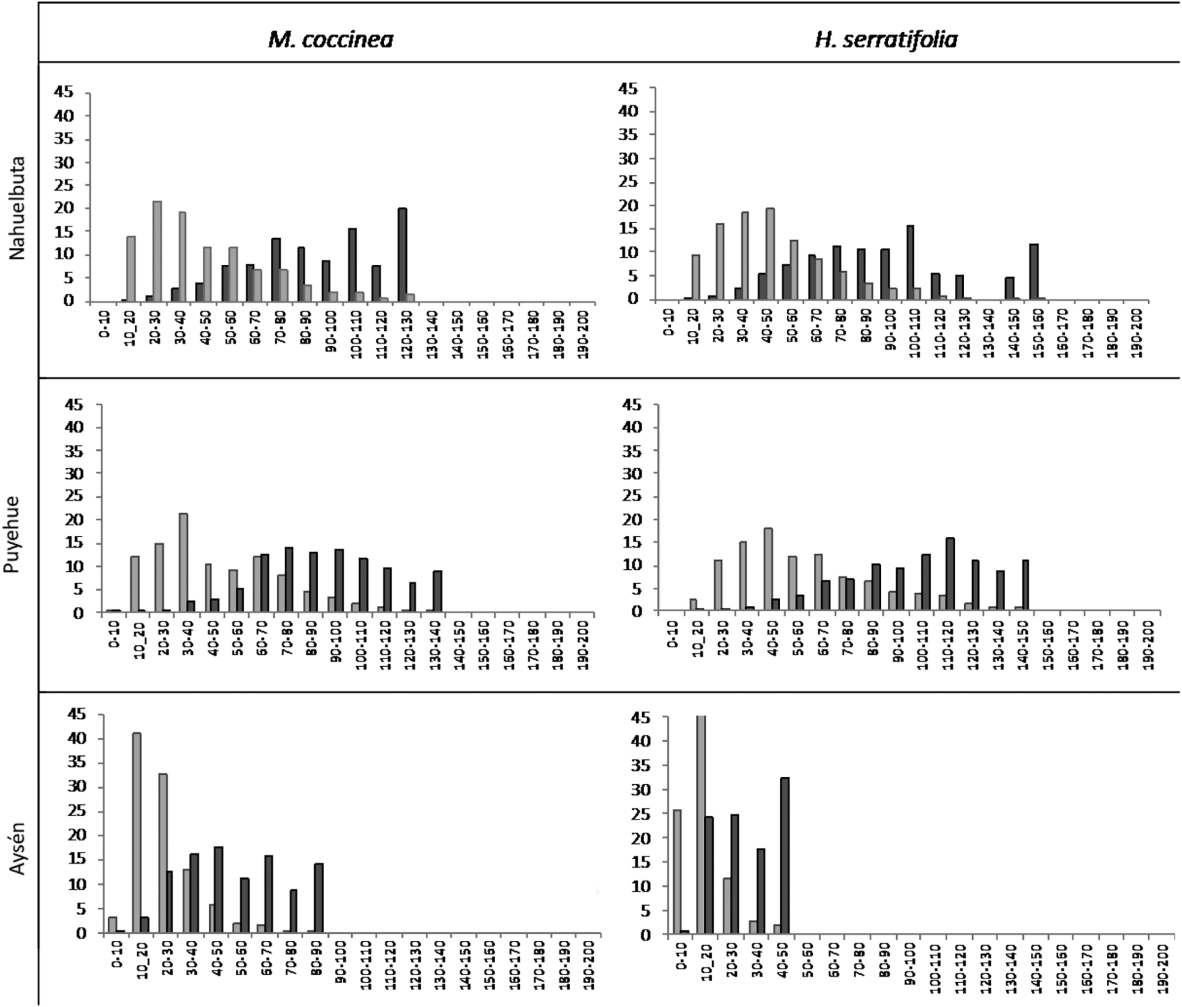
Frequency distribution of the diameter (μm) of xylem vessels (light gray) and their relative contributions to total hydraulic conductivity (dark gray) in two shared species at the study sites *Mitraria coccinea* (left) and *Hydrangea serratifolia* (right). The frequency distribution is significantly different for the diameter of xylem vessel and relative contribution to total hydraulic conductivity, as shown by the Kolmogorov–Smirnov test (Xylem vessel for the specie *Mitraria coccinea* sp., in Nahuelbuta *p* = .002, in Puyehue *p* = .01, and Aysén *p* = .001; relative contribution to total hydraulic conductivity for the specie *Mitraria coccinea* sp. for Nahuelbuta *p* = .01, Puyehue *p* = .013, Aysén *p* = .001; Xylem vessel for the specie *Hydrangea serratifolia* in Nahuelbuta *p* = .003, Puyehue *p* = .027, and Aysén *p* = .001; relative contribution to total hydraulic conductivity for the specie *Hydrangea serratifolia* in Nahuelbuta *p* = .04, Puyehue *p* = .022, and Aysén *p* = .001).

We observed a decrease in the proportion of wide vessels toward the cold extreme of the gradient (Figure 2, exemplifying two species along the latitudinal gradient). Considering that vessels with diameter higher than 100 μm are responsible for most of *K*_s_, we estimated that 14.7% of vessels are responsible for 69% of theoretical hydraulic conductivity in Nahuelbuta. Meanwhile, 7.6% of vessels are responsible for 47% of theoretical hydraulic conductivity in Puyehue. No species showed vessels with diameter higher than 100 μm in Aysén, and all hydraulic conductivity was generated by narrow vessels. The frequency distribution is significantly different for the diameter of xylem vessel and relative contribution to total hydraulic conductivity, as shown by the Kolmogorov–Smirnov test (Xylem vessel for the specie *Mitraria coccinea* sp., in Nahuelbuta *p* = .002, in Puyehue *p* = .01, and Aysén *p* = .001; relative contribution to total hydraulic conductivity for the specie *Mitraria coccinea* sp. for Nahuelbuta *p* = .01, Puyehue *p =* .013, Aysén *p* = .001; xylem vessel for the specie *Hydrangea serratifolia* sp. in Nahuelbuta *p* = .003, Puyehue *p* = .027, and Aysén *p* = .001; relative contribution to total hydraulic conductivity for the specie *Hydrangea serratifolia* sp. in Nahuelbuta *p* = .04, Puyehue *p* = .022, and Aysén *p* = .001). All these results show that the relative contribution of large vessel diameter to water transport was reduced along the latitudinal gradient, evidencing a trade‐off between efficiency and vascular security at the cold extreme.

**FIGURE 3:**
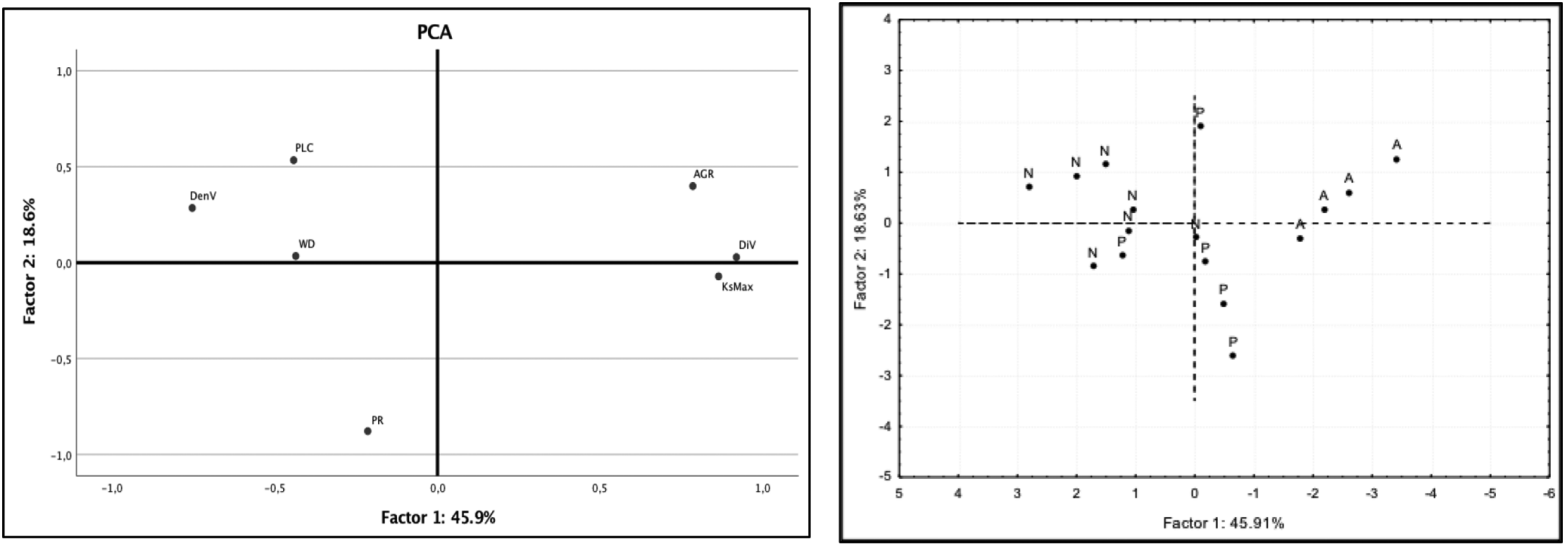
Principal Component Analysis (PCA) considering hydraulic traits, wood anatomy, and growth rate, in the three study sites. The study sites along the temperature gradient were N: “Nahuelbuta,” P: “Puyehue,” and A: “Aysén.” The first component explains 45.9% of variation and the second component 18.6%. For abbreviations, see main text.

**FIGURE 4:**
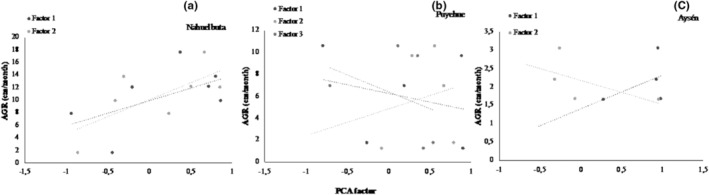
Linear regression between PCA factor obtained for each site and Apical Growth Rate for (a) Nahuelbuta (Factor 1 *R* ^2^ = .71, *p* = .017; Factor 2 *R* ^2^ = .57 *p* = .06), (b) Puyehue (Factor 1 *R* ^2^ = .007, *p* = .66; Factor 2 *R* ^2^ = .039, *p* = .75, Factor 3 *R* ^2^ = .14, *p* = .1) and (c) Aysén (Factor 1 *R* ^2^ = .21, *p* = .06; Factor 2 *R* ^2^ = .34, *p* = .07). Each point represents a score for each trait in the PCA analysis and mean above growth rate (AGR) of the species.

The Principal Component Analysis based on all functional traits studied shows that the first component explained 45.9% of the variation and the second component explained 18.6% of the variation. This PCA analysis shows that high performance is related to high efficiency in water transport (high specific hydraulic conductivity and wide vessels) (Figure 3). On the other side, low performance was related to low efficiency in water transport (high wood density, narrow vessels, and high percentage of loss conductivity). This relationship between performance, efficiency, and safety in water transport is robust enough to functionally differentiate liana communities between sites along the latitudinal gradient (Figure 3). The linear regression of the first and second components and the above growth rate (AGR) for each site was significant for Nahuelbuta (Factor 1 *R* ^2^ = .71, *p* = .017; Factor 2 *R* ^2^ = .57 *p* = .06; Figure 4), where traits that are positively associated with growth rate were the maximum specific hydraulic conductivity (*K*_s_max_) and xylem vessel diameter and density; on the contrary, the percentage of loss conductivity (PLC) and root pressure was negative related to growth rate. In the Puyehue data set, none of the components were significantly related to AGR (Factor 1 *R* ^2^ = .007, *p* = .66; Factor 2 *R* ^2^ = .039, *p* = .75, Factor 3 *R* ^2^ = .14, *p* = .1; Figure 4). For Aysen, a low and nearly significant linear regression (Factor 1 *R* ^2^ = .21, *p* = .06; Factor 2 *R* ^2^ = .34, *p* = .07; Figure 4) was observed between the first and second components. The traits that positively relate to growth rate were xylem vessel diameter, root pressure and wood density, and the percentage of loss conductivity was negatively related.

## 4 DISCUSSION

### 4.1 Liana performance, cold environment, and embolism

There was a strong decrease in liana performance toward the cold extreme of the latitudinal gradient, a consequence of the loss in water transport efficiency due to important levels of freeze–thaw embolism (Figure 1). Different studies in tropical forests have shown that the competitive advantage of lianas relies on a high growth rate, which allows them to outcompete trees and other growth forms (Isnard & Silk, 2009; Putz & Mooney, 1991; Schnitzer, 2018; Schnitzer & Bongers, 2002). However, in cold environments, this advantage is lost or at least significantly reduced; growth rate was five times lower in lianas at the colder site (Aysén) than lianas at the warm end of the gradient (Nahuelbuta) (Figure 1; Table 2). Moreover, growth rate was inversely correlated with PLC along the gradient (Figure 1), demonstrating that the reduction in performance is related to the vulnerability of their vascular system to freeze–thaw embolism.

Freeze–thaw embolism would restrict lianas' performance in two ways: first, it would generate a delay in the growth period because liana species would need to recover their water transport capacity before they start phenological processes or enter their growth period in early spring (Christensen‐Dalsgaard & Tyree, 2014; Ewers et al., 1997; Nardini et al., 2011). Therefore, they would need to invest more carbon into reverting embolism rather than allocating it to growth (e.g., via root pressure which is osmotically generated), as compared to species that have avoided freeze–thaw embolism. Second, species that lose a high percentage of their hydraulic conductivity due to embolism could permanently lose the functioning of their vascular system. In this case, lianas would depend on the generation of new xylem (Améglio et al., 2002; Ewers et al., 1991, 1997; Sperry et al., 1987), which, again, would limit resource allocation to growth. Either way, freeze–thaw embolism would severely limit the growth capacity of liana species because of limited water transport to the leaves and therefore a decrease in gas exchange and photosynthesis (McDowell et al., 2019). Therefore, freeze–thaw embolism would imply losses in plant performance and productivity in the long term.

Our results support the hypothesis that climbing habits are incompatible with cold environments. Although several authors have proposed this hypothesis in the last decades (Table S1), our results show evidence of a performance reduction in lianas species in natural environments because of their high vulnerability to embolism in cold environments.

### 4.2 Hydraulic transport, PLC, and wood anatomy

Water transport capacity (*K*_s_max_) of liana species was much less efficient in the cold extreme of the latitudinal gradient (Aysén) than in the warm extreme (Nahuelbuta) (Table 2). In fact, the maximum specific hydraulic conductivity (*K*_s_max_) in Puyehue and Aysén was 1.9 and 26 times lower than in Nahuelbuta, respectively. This loss of hydraulic conductivity is a critical point in liana species performance because a high hydraulic conductivity is needed to supply water to the large foliar area they develop (Isnard & Silk, 2009; Schnitzer & Bongers, 2002) and to support the high growth rates that characterize lianas compared to other growth forms (Putz & Mooney, 1991; Schnitzer, 2005). Thus, as shown by our results (Table 2), the loss of efficiency in water transport results in a lower ecological performance along the latitudinal gradient.

The decrease in specific hydraulic conductivity (*K*_s_max_) could be due to a joint effect between the selection of narrow vessel diameter (VD) and the increase in PLC toward higher latitudes (Table 2). Since wide vessels are highly prone to freeze–thaw embolism (Davis et al., 1999; Ewers et al., 1991; Gartner et al., 1990), they would not be functional toward higher latitudes (Jiménez‐Castillo & Lusk, 2013), so cold temperatures would be acting as a selection factor on narrow vessel diameters. We observed that liana species at higher latitudes have significantly decreased vessel diameters, which permit safety but not efficiency in water transport. The selection for narrow vessel diameter is also related to the observed increase in vessel density and wood density (Table 2), a response that has been documented in different growth forms (Chave et al., 2009; Martínez‐Cabrera et al., 2011; Poorter et al., 2010). In addition, the percentage of loss conductivity (PLC) increases from 25% to 37% from the warmer to the colder extreme of the latitudinal gradient. The PLC would exacerbate the loss of efficiency in water transport, for example, liana species lose 25% of their hydraulic conductivity in Nahuelbuta compared to the other sites (?) due to freeze–thaw embolism (PLC), which reduced their maximum specific hydraulic conductivity (*K*_s_max_) from 28.7 to 21.6 kg s^−1^ m^−1^ MPa^−1^ approximately. Meanwhile, liana species reduced 37.5% of their hydraulic conductivity in Aysén due to PLC, which reduced the conductivity from 1.1 to 0.68 kg s^−1^ m^−1^ MPa^−1^. Therefore, the total effect of PLC over *K*_s_max_ caused a reduction of 2.2 and 32 times in Puyehue and Aysén, respectively, as compared to Nahuelbuta. This supports the concept of the loss of efficiency of water transport– either by reduction of vessel diameter or elevated levels of embolism– which decreases the performance of lianas in cold environments.

### 4.3 Safety of water transport in cold environment

Cold temperatures would serve as strong selective pressure, leading plants to select vascular anatomy features that prevent the formation or spread of embolism (Brodersen & McElrone, 2013; Brodribb & Holbrook, 2004). When we analyzed the complete dataset of the frequency distribution of xylem vessel diameter and their relative contribution to total hydraulic conductivity, we observed that 50% of hydraulic conductivity is sustained by 11% of xylem vessels (>100 μm) (Figure 2), so losing the functionality of this small percentage of vessels would have a great impact on the conductivity of the individual. Lianas favor the safety of hydraulic transport in freeze conditions, showing up to three times reduction in vessels diameter with increasing latitude (Table 2), losing the diameter class responsible for the highest percentage of conductivity (Figure 2). Also, the selection of a high percentage of narrow vessels (~80%), associated with a slight increase in the density of the wood and xylem tissue made up of denser xylem vessels (Table 2) would favor the recovery of hydraulic conductivity, since they can remain functional after freeze events (Davis et al., 1999; De Guzman et al., 2016; Hacke & Sperry, 2001; Sperry & Sullivan, 1992). This selection could function as a backup system that delivers water to the leaves when large vessels are dysfunctional (Ewers, 1985; Sperry et al., 2006; Zwieniecki & Holbrook, 2009), which would be functionally relevant at the start of the growth season in temperate environments (Chiu & Ewers, 1992).

The decrease in vessel diameter, to a size as narrow as that observed in trees (Jiménez‐Castillo & Lusk, 2013), also decreases the hydraulic conductivity to such a magnitude that lianas would not be able to sustain a high ratio of leaf area/active xylem area or a high gaseous exchange (Davis et al., 1999). This, in turn, would lead them to lose their main competitive advantage, which is their high growth rate. So, this trade‐off between safety and efficiency of water transport would make their climbing habit less competitive and would sustain the mechanism behind the already‐observed pattern of a lesser diversity of liana species in cold climates (Lobos‐Catalán & Jiménez‐Castillo, 2019).

Root pressure would allow lianas species to recover their water transport capacity after freeze–thaw embolism occurs (Ewers et al., 1997; Sperry et al., 1987; Tibbetts & Ewers, 2000; Yin et al., 2018), a trait with adaptive value in cold environments (Jiménez‐Castillo & Lusk, 2013). All studied liana species generate root pressure, but we did not observe an increase toward higher latitudes (Table 2), and the magnitudes registered would not allow for reverting embolism along the whole stem of individuals that reach the forest canopy. In other studies, it has been frequently observed that root pressure is too low for reverting completely the embolism (Ewers et al., 1997; Tibbetts & Ewers, 2000). Only in particular cases, species of genus *Cissus* have shown root pressure that would dissolve embolism up to 14–20 meters high in the plant (Fisher et al., 1997), but we did not observe such patterns in the *Cissus* species included in our study. Our results indicate that the root pressure would be enough only to fill vessels of the root system and the basal part of the sprouted stems (Ewers et al., 1997; Isnard & Silk, 2009). Therefore, root pressure would be a first step to achieving the recovery of conductivity, as it could be associated with other recovery traits such as the increase in vascular tension by transpiration “throw‐away strategy” (Hacke & Sperry, 2001; Nardini et al., 2011) and/or the generation of new vessels in early wood (Sperry & Sullivan, 1992; Tibbetts & Ewers, 2000). Although root pressure could be an important trait for lianas that inhabit cold environments, more studies are needed to estimate its actual contribution to the recovery of hydraulic conductivity.

### 4.4 Trade‐off between the efficiency and safety of water transport, overview

Our results show that lianas sustain a high growth rate in habitats with infrequent frost events, sustained by the efficiency of their water transport systems, resulting from a set of traits: wide xylem vessels, low vessel density, and high specific hydraulic conductivity (Figures 2 and 3). While, in habitats with frequent frost events, at higher latitudes, liana species suffer a strong decrease in their growth rate caused by elevated levels of PLC, narrow xylem vessel diameter, high vessel density, high wood density and (Table 2). These functional strategies that associate performance with the efficiency and safety of water transport are strong enough to functionally differentiate liana communities along the latitudinal gradient (Figure 2), and support the functional mechanism that underlies the lower diversity of lianas species in temperate ecosystems (Lobos‐Catalán & Jiménez‐Castillo, 2019), explaining the variation in the performance of liana species along this latitudinal temperature gradient and providing evidence for the “cold hypothesis” in natural environments.

Other studies in tropical ecosystems have shown that the trade‐off in efficiency and safety of water transport in lianas is decoupled when the environmental pressure is caused by drought embolism (De Guzman et al., 2016; van der Sande et al., 2019). Unlike trees, lianas only favor efficiency and have not changed in their safety traits. So, this trade‐off between efficiency and safety of water transport would change depending on the environmental pressure or the ecosystem that the lianas inhabit. Moreover, different authors show that it is impossible to combine high hydraulic efficiency and high safety (van der Sande et al., 2019). Species with high efficiency in water transport also have high gas exchange rates, which contribute to fast resource acquisition and growth. Species with high safety in water transport have low gas exchange rates and other traits associated with a conservative life history strategy of resource conservation and slow growth (Santiago et al., 2004). These latter functional strategies would be those observed in liana species studied at the extremes of the latitudinal gradient.

Although our study was conducted at the local scale, it speaks to a variation in liana species performance along a latitudinal temperature gradient and the relationship of this performance with hydraulic functional traits, while also contributing to an understanding of the different mechanisms that explain liana diversity patterns. After presenting our results, we want to propose the idea that mixed mechanisms would explain the liana diversity patterns. On the one hand, it has been documented that high growth rate is the main competitive ability of lianas in tropical ecosystems and that this is augmented in sites with low rainfall and/or high seasonality rainfall (Dewalt et al., 2000, 2006, 2015; Schnitzer, 2005; Schnitzer & Bongers, 2002, 2011). On the other hand, temperature is the main variable explaining the patterns of liana richness and abundance in temperate ecosystems (Jiménez‐Castillo et al., 2007; Lobos‐Catalán & Jiménez‐Castillo, 2019) because of its effect on performance reduction. Consequently, liana diversity patterns would be the result of the differential influence of environmental factors and mixed explanatory mechanisms. However, more studies are needed in order to prove this proposal, particularly studies on the functional mechanisms behind diversity patterns of lianas in different ecosystems.

Through a functional approach, the performance of the liana species has been linked to the underlying mechanisms involved based on functional hydraulic strategies. These results allow us to explain how temperature tracks liana diversity patterns along a latitudinal gradient within the temperate rainforest of South America. This study also contributes to the representation of this group of plants in functional trait biology, which is a tool that can be applied in the fields of conservation, forest dynamics, and response to global climate change.

## AUTHOR CONTRIBUTIONS

**Paulina Lobos‐Catalán:** Conceptualization (equal); data curation (equal); formal analysis (equal); investigation (equal); methodology (equal); resources (equal); validation (equal); visualization (equal); writing – review and editing (equal). **Mylthon Jiménez‐Castillo:** Conceptualization (equal); funding acquisition (equal); project administration (equal); writing – original draft (equal); writing – review and editing (equal).

## FUNDING INFORMATION

The first author was supported by CONICYT doctoral research grant number 21110389 and MJC by FONDECYT 1130898.

## CONFLICT OF INTEREST STATEMENT

The authors declare that they have no conflicts of interest.

## Supporting information


Tables S1–S3

## Data Availability

Hydraulic traits data of lianas, for each site and by species, is available at https://www.try‐db.org/TryWeb/542_Lianahydraulics.xlsx.
