# Supplementary material for: The functional mechanism behind the latitudinal pattern of liana diversity: Freeze–thaw embolism reduces the ecological performance of liana species
Source: Ecol Evol. 2023 Sep 19;13(9):e10486. doi: 10.1002/ece3.10486 (PMC10509155; doi:10.1002/ece3.10486)
Supplement: Supplementary file 1 — Tables S1–S3 [file ECE3-13-e10486-s001.docx]

**Supplementary information**

**Table S1:** Publication that state the "Cold hypothesis" as modeler of GPLD, highlighting the works that present empirical evidence (Y = Yes or N = No) understood as the quantitative evaluation of the proposed hypothesis and the cited articles related to the same hypothesis.

| **Author** | **Year** | **Article** | **Cold hypothesis** | **Empirical data** | **Cited articles** |
| --- | --- | --- | --- | --- | --- |
| Zimmermann | 1971 | Trees: Structure and function | Y | N |  |
| Ewer | 1985 | Xylem structure and water conductions in conifer trees, dicot trees, and lianas. | Y | N |  |
| Sperry, Holbrook, Zimmerman & Tyree | 1987 | Spring Filling of Xylem Vessels in Wild Grapevine | N | N |  |
| Tyree & Sperry | 1989 | Vulnerability of xylem to cavitation and embolism | Y | N |  |
| Ewers, Fisher & Chiu | 1990 | A Survey of Vessel Dimensions in Stems of Tropical Lianas and Other Growth Forms | N | N |  |
| Cochard & Tyree | 1990 | Xylem dysfunction in Quercus: vessel sizes, tyloses, cavitation and seasonal changes in embolism | N | N |  |
| Gartner | 1990 | Water transport properties of vines and trees stem in tropical deciduous forest | N | N |  |
| Ewers, Fisher & Fichtner | 1991 | Water flux and xylem structure in vines. | Y | N |  |
| Gentry | 1991 | The distribution and evolution of climbing plants | N | N |  |
| Sperry & Sullivan | 1992 | Xylem embolism in response to freeze-thaw cycles and water stress in ring-porous, diffuseporous,and conifer species | N | N |  |
| Sperry, Nichols, Sullivan & Eastlack | 1994 | Xylem Embolism in Ring-Porous, Diffuse-Porous, and Coniferous Trees of Northern Utah and Interior Alaska | Y | N |  |
| Tyree & Ewers | 1996 | Hydraulic architecture of woody tropical plants. | N | N |  |
| Fisher, Angeles, Ewers, Lopez | 1997 | Survey of root pressure in tropical vines and woody species | Y | N | Sperry et al 1987 |
| Ewers, Cochard & Tyree | 1997 | A survey of root pressures in vines of a tropical lowland forest | Y | N | Cochard & Tyree 1990; Sperry & Sullivan 1992; Sperry et al 1994 |
| Davis, Sperry & Hacke | 1999 | The relationship between xylem conduit diameter and cavitation caused by freezing | Y | N |  |
| Tibbets & Ewers | 2000 | Root pressure and specific conductivity in temperate lianas | Y | N | Ewers1985; Sperry & Sullivan 1992 |
| Schnitzer & Bonger | 2002 | The ecology of liana and their rol in the forest | Y | N | Tyree, M. and Ewers, F.W. (1996) |
| Bhattarai & Vetaas | 2003 | Variation in plant species richness of different life forms along a subtropical elevation gradient in the Nepal | N | Y |  |
| Molina Freaner, Castillo, Tinoco & Castellanos | 2004 | Vine species diversity across environmental gradients in northwestern Mexico | Y | N | Ewers 1985;Sperry&Sullivan 1992 |
| Schnitzer | 2005 | A Mechanistic Explanation for Global Patterns of Liana Abundance and Distribution | Y | N | Sperry et al. 1987; Ewers et al. 1991, 1997 |
| Jiménez-Castillo & Lusk | 2007 | Elevational parallels of latitudinal variation in the proportion of lianas in woody floras | Y | Y | Gentry 1991 |
| Isnard & Silk | 2009 | Moving with climbing plants from Charles Darwin in time into the 21st century | Y | N | Gentry, 1991 ; Schnitzer, 2005 ; Jiménez-Castillo et al., 2007 |
| Hu, Li & Li | 2010 | Geographical and environmental gradients of lianas and vines in China | Y | N | Zimmermann 1971; Gentry 1991;Ewers et al 1997; Davis et al 1999; Tibbetts & Ewers 2000;Jiménez-Castillo 2007 |
| Gallagher, Leishman & Moles | 2011 | Traits and ecological strategies of Australian tropical and temperate climbing plants | Y | N | Jiménez & Lusk 2007 |
| Schnitzer & Bonger | 2011 | Increasing liana abundance and biomass in tropical forests | Y | N | Sperry et al. 1987; Schnitzer 2005 |
| Jiménez-Castillo & Lusk | 2013 | Vascular performance of woody plants in a temperate rainforest | Y | N |  |

**Table S2:** List of species included on this study, climbing habit, family, latitudinal distribution and studied site were each species was present (N= Nahuelbuta, P=Puyehue, A=Aysén) it is mentioned.

| **Species** | **Family** | **Climbing habit** | **Site** | **Latitudinal distribution** |
| --- | --- | --- | --- | --- |
| *Boquila trifoliolata* | Lardizabalaceae | Twiners | N- P | 35.08° - 42.42°S |
| *Cissus striata* | Vitaceae | Tendril climbers | N-P | 33.5° - 46.4°S |
| *Elytropus chilensis* | Apocynaceae | Twiners | P | 34.4° - 43.2S |
| *Hydrangea serratifolia* | Hydrangeaceae | Adventitious roots | N-P-A | 32.5° - 46.4°S |
| *Mitraria coccinea* | Gesneriaceae | Adventitious roots | N-P-A | 30.4° - 50.1°S |
| *Campsidium valdivianum* | Bignoniaceae | Twiners | A | 35.4° - 50.2°S |
| *Muehlenbeckia hastulata* | Polygonaceae | Twiners | N | 30.3° - 42.5°S |
| *Berberidopsis corallina* | Berberidopsidaceae | Twiners | N | 35.5° - 40.1°S |
| *Griselinia ruscifolia* | Griseliniaceae | Adventitious roots | A | 38.2° - 46.4°S |
| *Lardizabala biternata* | Lardizabalaceae | Twiners | N | 32.3°- 40.9°S |

**Table S3**: Generalized linear mixed models and their corresponding value of the ΔDIC. We evaluated each model with and without the effect of phylogeny, from which we calculated ΔDIC. We considered that ΔDIC <10 showed no significant effect of the phylogeny. Sites (Nahuelbuta, Puyehue, Aysén). Where AGR: growth rate, k_s_ = maximum specific hydraulic conductivity, PLC= loss of conductivity due to embolism, V_D_ = average vessel diameter, WD = wood density

| Model | ∆DIC |
| --- | --- |
| AGR ~ site | 0.1444 |
| k_s _max_~ site | 0.0214 |
| PLC ~ site | -0.0669 |
| P_x_ ~ site | 0.0863 |
| WD~ site | 0.5955 |
| V_d_~ sitio | 0.1318 |
| Vden~ sitio | 0.0087 |
| AGR ~ PLC | 0.663 |
| AGR ~k_s_*PLC | -0.0077 |
| AGR ~k_s_+ PLC | 0.7547 |
| AGR ~V_d_ | 0.8469 |
| AGR ~ WD | 1.092 |

We evaluate the effect of phylogeny on variation in functional traits, we used Bayesian Mixed Linear Models implemented in the package MCMCglmm v1.10 (Hadfield & Nakagawa, 2010) in R Core Team (2020). This analysis allows to include phylogeny as a covariance matrix of random effects in a mixed linear generalized model (GLMM). We obtained this matrix based on the phylogenetic tree of the focal species which we constructed from the phylogenetic distance between their genera. We compared models that include the phylogenetic covariance matrix as a random factor with a model without the phylogenetic factor. In all MCMCglmm analysis we used 110000 iterations, with 10000 burning, and 100 intervals with not informative priors. In the prior, the residual variance was set to 1 with v = 0.002, as recommended in Hadfield (2014) for a Gaussian distribution. We compared models with and without phylogeny using the Deviance Information Criterion (DIC) between them (Spiegelhalter et al., 2002). We used the recommended limit of ΔDIC> 10 to reject the null hypothesis (Hadfield & Nakagawa, 2010).
